# Variations of insecticide residual bio-efficacy on different types of walls: results from a community-based trial in south Cameroon

**DOI:** 10.1186/1475-2875-10-333

**Published:** 2011-11-02

**Authors:** Josiane Etang, Philippe Nwane, Jean Arthur Mbida, Michael Piameu, Blaise Manga, Daniel Souop, Parfait Awono-Ambene

**Affiliations:** 1 Laboratory of Medical Entomology, Organisation de Coordination pour la lutte contre les Endémies en Afrique Centrale, P. O. Box 288 Yaoundé, Cameroon; 2 Faculty of Medicine and Pharmaceutical Sciences, The University of Douala, P. O. Box 2701 Douala, Cameroon; 3 Faculty of Science, The University of Yaounde I, P. O. Box 818 Cameroon; 4 Faculty of Science, The University of Douala, P. O. Box 7064 Bassa-Douala, Cameroon; 5 Centre Supérieur des Sciences de la Santé, Université catholique d'Afrique Centrale BP 1110 Yaoundé, Cameroun; 6 Division of Health Promotion, Ministry of Health, Cameroon; 7 Service of Pesticide regulation, Ministry of Agriculture and rural development, Cameroon

**Keywords:** Insecticides, indoor residual spraying, malaria vector control, Cameroon

## Abstract

**Background:**

Determination of residual activity of insecticides is essential information for the selection of appropriate indoor spraying operation. The present study was undertaken to evaluate the residual effect of three candidate insecticide formulations on different indoor surfaces in order to guide future interventions, in the context of Cameroon and other African countries.

**Methods:**

The study was conducted in the Ntougou neighbourhood in Yaoundé (capital city of Cameroon). Bendiocarb WP, lambda-cyhalothrin CS and deltamethrin WG were sprayed on the indoor wall surfaces of local cement, wood and mud houses. Their effects on the knockdown and mortality of the Kisumu susceptible strain of *Anopheles gambiae *s.s were assessed each month from March to September 2009, using the WHO plastic cones test. Knockdown and mortality rates were compared between different surfaces using Chi-square test. A Kaplan-Meir model was used to estimate the time of treatment failure.

**Results:**

With bendiocarb WP, the knockdown rates were frequently above 98% during 13 weeks after spraying, except on mud walls where it significantly decreased at the 13^th ^week (P < 0.05). With lambda cyhalothrin CS, the knockdown rates remained 100% on wood surfaces during the 26 weeks trial. However, it significantly decreased on concrete and mud surfaces from the 11^th ^(83%) and the 20^th ^(88%) weeks respectively (P < 0.05). With deltamethrin WG, it remained high on concrete surfaces during 26 weeks (> 98%); while it varied between 60 and 100% on wood or mud surfaces. The survival estimates of bendiocarb WP treatments remaining effective in killing *An. gambiae *s.s. (mortality rate ≥ 80%) was > 13 weeks on cement and wood surfaces and 13 weeks on mud surfaces. Those of lambda-cyhalothrin CS were > 26 weeks on wood surfaces, and 20 weeks on concrete and mud surfaces. By contrast, those of deltamethrin WG were 26 weeks on concrete, 20 weeks on mud surfaces and 15 weeks on wood surfaces.

**Conclusion:**

Current data suggest variable durations of spray cycles for each product, according to the type of wall surfaces, highlighting the importance of testing candidate products in local context before using them in large scale.

## Background

Indoor residual spraying (IRS) is one of the effective strategies against anopheline, such as *Anopheles gambiae *s.l. and *Anopheles funestus*, the main malaria vectors in Africa [1,2]. In 2007-2009, some countries (Botswana, Namibia, South Africa, and Swaziland) achieved ≥ 50% reduction in malaria cases by reaching > 70% coverage of IRS [3]. Coverage of IRS is indeed increasing, but there is need to assess how far it is reaching the targeted populations and where else it would have added effect. In addition, a question mark hangs over their long-term effectiveness. In parts of Africa where infrastructure is especially weak, universal vector control coverage may not be achieved with IRS alone, and LLNs will continue to be needed to achieve and sustain this goal.

Identifying an appropriate and sustainable vector control strategy is therefore a major step toward achieving universal coverage of interventions, as emphasized in the Global Malaria Action Plan (GMAP) and contributing to millennium development goals (MDGs) targets 4, 5, and 6 [3]. This requires understanding the relationship between the available tools and environmental or socio-economic factors that can affect the effectiveness of interventions. Such factors are manifold, but a major distinction can be made between intrinsic and extrinsic factors. Intrinsic factors may be defined as characteristics belonging to the intervention itself, while extrinsic factors are mostly part of the environment or linked to human behaviour and living conditions (socio-economic factors). As for vector control insecticide-based interventions, intrinsic factors include insecticide formulation, mode of action, dosage, properties (including knockdown, killing, exito-repellent effects) and type of treatment (IRS, LLINs, Sheets) [4,5]. Extrinsic factors which include physical and biological factors mostly affect the development and survival of the mosquito (behavior, resistance to insecticides, temperature, humidity, etc) [6,7], while human activities, behavior and living conditions (acceptability, accessibility, rate of coverage) may provide an additional risk of intervention failure or success [8]. Understanding and considering environmental, socio-economic and other factors that can jeopardize the effectiveness of malaria interventions should be given due considerations in the African context and especially when dealing with communities at different levels of incomes and living conditions.

In Cameroon, malaria is the primary cause of mortality and morbidity in health centres [9]. Since year 2008, the National Malaria Control Programme (NMCP) has been scaling up long-lasting insecticidal nets (LLINs) throughout the country, to reduce the contact between malaria vectors and human hosts. IRS is considered as an alternative strategy to complement LLINs and achieve vector control universal coverage, especially in areas where *An. gambiae *s.l. has developed resistance to pyrethroids [10,11]. Apart from a few pilot operations, IRS is not implemented because of high transaction costs and lack of data for proper planning and decision.

In this context, the present study was performed aiming at providing information to guide spraying actions. The objective was to assess the duration of residual effect of three candidate IRS products: bendiocarb WP, lambda-cyhalothrin CS and deltamethrin WG, on knockdown and mortality of *An. gambiae *s.s. in different types of human dwellings usually found in Cameroon, in order to guide future IRS operations.

## Methods

### Trial site

The field operation was carried out in the semi urban area of Ntougou neighbourhood in Yaoundé (capital city of Cameroon) from March to September 2009. Ntougou is characterized by the equatorial climate, with many *An. gambiae *and other culicinae breeding sites around the vegetable plots. *An. gambiae *s.s. is present all year-round and is the main malaria vector in this area (Etang, unpublished data). There was no recent history spraying in the area. The study was performed in normal occupation conditions of the houses so the results would express the action of insecticides in real field use conditions.

### Study design

A total of 39 dwellings of two to three rooms were enrolled in the study, e.g. 13 dwellings with walls made of unpainted mud, 13 dwellings with walls made of unpainted wood, 13 dwellings with walls made of concrete blocks covered with cement and painted. Among each batch of 13 dwellings, four were sprayed with FICAM^® ^VC (bendiocarb wettable powder 80%), four with ICON 10 CS (lambda-cyhalothrin Capsule Suspension 100 g/l), four with K-Othrine WG 250 (deltamethrin water dispersible granule 250 g/kg) and the last one with tap water as control. Mixtures of water and commercial products were applied to internal walls of houses, at operational dosages of deltamethrin 0.02 g a.i/m^2^, lambda-cyhalothrin 0.025 g a.i/m^2 ^and bendiocarb 0.4 g a.i/m^2 ^as recommended by WHOPES [12], each in one day, for three days in a row. Sprays were applied using a compression sprayer (Micron-air CS 10) fitted with 8002 flat fan nozzles. The flow rate was 0, 75l per minute with a pressure of 3 bars. During spays, the lance were maintain at 45 cm from the wall. Pre-dosed sachets of insecticide were used to obtain mixtures recommended in the sprayer. Peoples living in the houses were asked to remain outside for three hours before re entering the treated houses.

For each of the three insecticides, nine rooms with cement walls, nine rooms with wood walls and nine rooms with mud walls were randomly chosen for follow-up. Bio efficacy of IRS was assessed one week after treatment and then every month during 3-6 months, in a total of 27 (3 × 9) treated rooms and three control rooms.

### Bioassays

The Kisumu reference strain of *An.gambiae *s.s. was used for bioassays. This strain originated from Kenya has been colonized for many years in the Laboratory of Medical Entomology (25 ± 2°C and 70-80% RH) in "Organisation de Coordination pour la lutte contre les Endémies en Afrique Centrale (OCEAC, Yaoundé, Cameroon)" and is free of any detectable resistance mechanisms [13]. Bioassays were carried out according to WHO protocol [14], using three to five days old non blood-fed female mosquitoes provided from the OCEAC insectary. Five WHO cones were fixed firmly on walls. Five mosquitoes were introduced in each cone by using a plastic aspirator. After 30 minutes exposure to treated wall, mosquitoes were transferred in white plastic labelled cups covered with untreated netting, and the knockdown rate (KD) was recorded 60 minutes post exposure. Then, mosquitoes were kept in the insectary and supplied with 10% sugar solution. Mortality rates were recorded after 24 hours holding period. Ten batches of five mosquitoes were used for each room, and 10 batches were exposed to control room sprayed with tap water. Bioassays were carried out at 25 ± 2°C temperature and 70-80% RH humidity.

### Data analysis

Knock down and mortality rates were calculated and analysed according to World Health Organization [14] to determine whether IRS was effective. Treatment was considered effective when mortality rate in exposed mosquitoes was > 80% and Knockdown rates > 95%. A Kaplan-Meir model using SPSS software, version 11.5 (SPSS Inc. 2002) was used to estimate the time of IRS failure. Knockdown and mortality rates were compared between different surfaces using Chi-square test. A p-value of < 0.05 was considered significant.

### Informed consent and ethical approval

All heads of households were informed about the study prior to initiation. Quarter leaders helped create awareness of the study within the community. The head of each household was asked to sign the consent form for their household to participate in this evaluation. Community members were informed that participation in the study was completely voluntary and that they may withdraw from the study at any time without penalty. This study was approved by the Ministry of Health Review Board in Cameroon.

## Results

A total of 1,500 female mosquitoes of the Kisumu *An. gambiae *s.s. susceptible strain was used each month for bio assays.

### Control assays in dwellings sprayed with tap water

No knockdown effect was observed after exposure of mosquitoes to control walls. The mortality rates recorded was always below 2%.

### Bio efficacy of insecticide indoor residual spraying

Based on the availability of the progeny of the Kisumu *An. gambiae *s.s. susceptible strain, bio efficacy data were recorded during weeks 2, 5, 8 and 13 for bendiocard WP house spraying; then during weeks 2, 6, 11, 15, 20 and 26 for lambda cyhalothrin CS and deltamethrin WG house spraying. No side effect of IRS was reported on inhabitants, workers or animals.

### Knockdown rates

**Figure 1:**
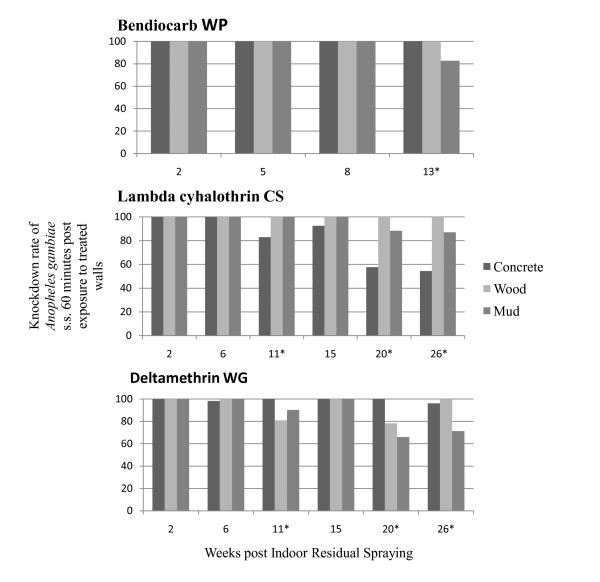
**Knockdown rates of susceptible *Anopheles gambiae *s.s. post contact with different insecticide sprayed walls**. *: significant difference between types of walls.

Variations of knockdown rates of the Kisumu susceptible strain of *An. gambiae *s.s. after contact with concrete, wood and mud sprayed surfaces are presented in Figure 1. Different patterns of knockdown rates were recorded with the three types of walls, depending on insecticide formulations and ages of the spray deposit.

With bendiocarb WP, the knockdown rates remained above 98% during eight weeks after spraying with, no matter the type of walls. At the 13^th ^week post-treatment, the residual knockdown rate on cement and wood walls was still 100%, while that of mud walls significantly decreased (P < 0.05). With lambda cyhalothrin CS, the knockdown rates remained 100% on wood surfaces during the 26-week trial. However, a rapid decrease was observed on concrete surfaces from the 11^th ^week (83%) to the 26^th ^week (54%), while the decrease on mud surfaces appeared on the 20^th ^weeks post treatment (88%) until the 26^th ^week (87%). With deltamethrin WG, the best duration of knockdown effect was recorded on concrete surfaces which displayed > 96% rates during 26 weeks. On wood and mud surfaces, the knockdown rates varied between 60 and 100%, especially from the 11^th ^to the 26^th ^weeks.

### Mortality rates and IRS survival estimates

**Figure 2:**
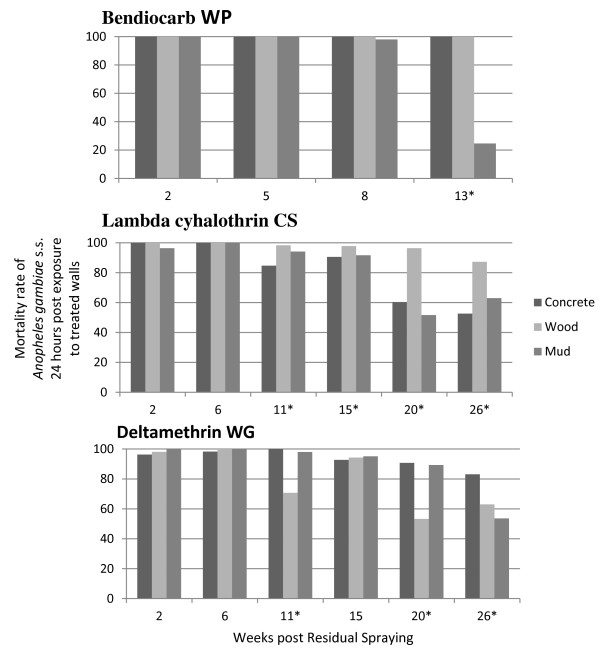
**Mortality rates of susceptible *Anopheles gambiae *s.s. post contact with different insecticide sprayed walls**. *: significant difference between types of walls.

**Figure 3:**
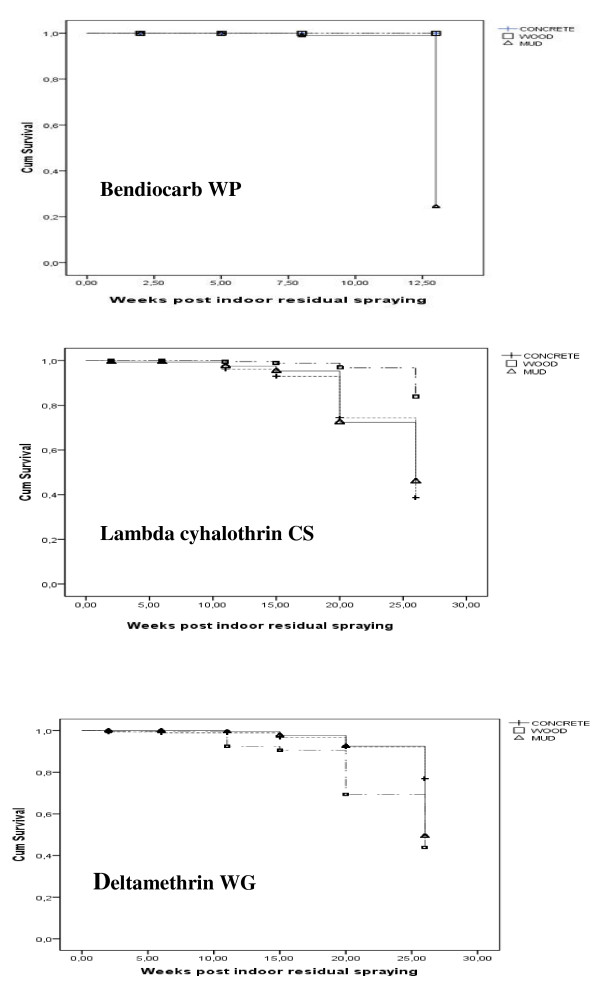
**Kaplan-Meier survival estimates of wall treatments remaining effective in killing susceptible *Anopheles gambiae *s.s**.

The mortality rates and the survival estimates of treatments remaining effective in killing the Kisumu susceptible strain of *An. gambiae *s.s. on concrete, wood and mud sprayed surfaces are presented in Figure 2, Figure 3 and Table 1 respectively. With bendiocarb WP, the mortality rates were mostly 98-100% during the 13 weeks assessment. No significant difference was observed between the three different surfaces (P > 0.6), except on mud surfaces at the 13^th ^week where the mortality rates dropped to 25%. Indeed, the survival estimates were > 13 weeks on cement and wood surfaces and 13 weeks on mud surfaces.

**Table 1:** Predictable duration of residual bio-efficacy of insecticide indoor spraying on different types of walls

Insecticide compounds formulations (1) and operational dosages	Estimated duration of residual bioefficacy (weeks)		
	
	Cement	Wood	Mud
Bendiocarb WP 0.4 g a.i/m^2^	> 13	> 13	13
Lambda-cyhalotrin CS 0.025 g a.i/m^2^	20	> 26	20
Deltamethrin WG 0.02 g a.i/m^2^	> 26	15	20

WP: wettable powder, CS: Capsule Suspension, WG water dispersible granule

With lambda-cyhalothrin CS, the mortality rates remained high on wood surfaces during the whole 26-week trial, ranging from 87% to 100%. However, a significant decrease was observed at the 20^th ^on concrete and mud surfaces, where it ranged from 50% to 60% (P < 0.05). The survival estimates were > 26 weeks on wood surfaces, and 20 weeks on concrete and mud surfaces. With deltamethrin WG, the mortality rates remained high on concrete and mud surfaces during the 20 first weeks of the trial, ranging from 90% to 100%, while it significantly decreased on wood surfaces early at the 15^th ^weeks (P < 0.05). On the 26^th ^week, the three surfaces were subject to a significant decrease of mortality rates (P < 0.05), although it was still > 80% on concrete surfaces while it dropped to less than 65% on wood and mud surfaces. The survival estimates of the treatment were 26 weeks on concrete, 20 weeks on mud surfaces, and 15 weeks on wood surfaces.

## Discussion

The first consideration to choose the insecticide to be used for IRS should is its proven effectiveness on the target vector species and its safety for inhabitants, workers, animals, and environment. In addition to the susceptibility of target species to insecticides, the duration of residual effect of insecticides is essential information. The importance of a more precise definition of the duration of the residual effect is in the need for programming cycles so that the human population remains protected until a new spraying is conducted.

Among the 12 insecticides recommended by WHO for residual indoor spraying against malaria vectors, are the three products used in this trial (Bendiocarb WP 80%, Lambda-cyhalothrin 10 CS and Deltamethrin WG 25%), all of them with residual activity estimated between two and six months [12]. Such a variation of the time makes it difficult to plan field activities, including the amount of product to be bought and the need to better define the cycles. To make progress, existing methods will have to be deployed more effectively. In many countries, malaria occurs mostly in the poorest, rural sectors of society, and even relatively simple control methods are rarely applied effectively.

In Cameroon, the first experience with IRS happened in southern equatorial and northern tropical parts of the country during the 1950s in the framework of malaria eradication pilot campaigns [15,16]. IRS in the south with DDT and dieldrin was a complete success (plasmodic index dropped below 1%). Unfortunately, dieldrin resistance of *An. gambiae *s.s. hampered this programme, which was stopped in 1960. The north was first sprayed with dieldrin, then with DDT in 1959 when dieldrin resistance emerged in *Anopheles arabiensis*. But after two years of spraying, the plasmodic index remained high (35%) and the programme stopped. The comparison between the success in the South and the failure in the north underlined the need of diversifying strategies according to a number of factors, including epidemiology of the disease, ecology and susceptibility of vectors, as well as human living conditions [17]. Use of insecticide-treated bed nets (ITNs), and then LLINs for large-scale malaria prevention has been promoted until now, whereas historically indoor residual spraying (IRS) was the primary intervention.

In the present study, the effectiveness of three insecticide formulations available at country level and that could be used for IRS was assessed, to supplement LLINs and achieve vector control universal coverage. Regarding the type of walls, variable durations of residual bioefficacy were observed for each insecticide, although all of them were within the range of two-six months reported in WHO recommendations [12]. Based on these data, the spraying cycles may not exceed 13 weeks for bendiocarb WP on mud walls while it may last 13 weeks at least for the others types of surfaces, 15 weeks for deltamethrin WG on wood walls, 20 weeks for lambda-cyhalothrin CS on cement and mud walls or deltamethrin WG on mud walls, and above 26 weeks for lambda-cyhalothrin CS on wood walls or deltamethrin WG on cement. The survival estimates of bendiocarb on concrete and wood surfaces do not clearly define the duration of spray cycles, due to the shortage of the observations. However the given information is helpful for decision making when considering the drastic drop of bendiocarb bio efficacy on mud surfaces at the 13^th ^week compared with wood and concrete surfaces. Variation in the residual efficacy of insecticide treated surfaces against *Triatoma infestans *was also noticed by other authors [18,19]. The persistency of insecticides, as revealed by mortality, depended on the type of surface, the dosage, and the age of spray deposits [20].

Bendiocarb WP showed shorter persistence (13 weeks) when applied to mud walls. One of the main reasons for the loss of insecticide activity may be the fast absorption by porous surfaces. Mud surfaces are very porous and the application of alkaline substances may degrade the molecule of the insecticide faster [21]. One- or- two-month residual effect was recorded with Lambda-cyhalothrin WP, compared with other pyrethroids (three-four months residual activity) in killing Brazilian malaria vectors (including *Anopheles albitarsis *s.l., *Anopheles triannulatus*, *Anopheles darling*i), emphasizing the need for shorter application cycles [21]. Nevertheless, the performance of Bendiocarb WP recorded during the current study on mud walls is similar to that reported on deltamethrin suspension concentrate (SC) and etofenprox WP against Brazilian malaria vectors. After four months experiment of indoor residual spraying treatments in experimental huts in Benin, bendiocarb was shown to be effective in controlling pyrethroid-resistant Anopheles, as well as fenitrothion and chlorpyriphos-deltamethrin mixture [22]. They were considered as effective alternatives to pyrethroids for indoor residual spraying against pyrethroid resistant malaria vectors. Bendiocarb decayed in less than four months, showing a short-life on cement walls, but was still considered as a promising insecticide to control resistant vectors. The authors suggested that a micro-encapsulation formulation of bendiocarb would make it last longer on treated surfaces.

Among the insecticides tested in this study, those which presented greater performance even on mud walls were deltamethrin WG and lambda cyhalothrin CS. Study on residual efficacy of deltamethrin 2.25% WG at 25 mg/m^2 ^against *Anopheles culicifacies *in India showed 100% mortality up to 12, 10, 9 and 12 weeks on mud, cement, brick and thatch surfaces respectively [23]. A village scale trial of deltamethrin 2.25% WG at 25 mg/m^2 ^against both anophelinae and culicinae mosquitoes also indicated a residual life about 12 weeks both on mud and cement plaster surfaces in India [24]. In Brazil, residual activity of SC formulation of deltamethrin at 25 mg/m^2 ^reported three, two and three months on wood, plastered brick and brick surfaces respectively [25]. The extended field trial of deltamethrin 2.5% WP at 25 mg/m^2 ^confirmed the long residual effectiveness from 15 to 16 weeks on both mud and cement plastered surfaces in India [26]. The residual activity of WG formulation of deltamethrin at 25 mg/m^2 ^was effective for six weeks after treatment on *Aedes *vectors in Kuala Lumpur, based on biweekly bioassay [27]. In Iran, deltamethrin WG 25% 25 mg/m^2 ^was reported to remain effective against a lab-bred *Anopheles stephensi *strain for three months on plaster surfaces, two-two-and-half months on mud cement or wood surfaces [28]. Persistence of effectiveness of ICON 10 CS has also been estimated up to two-four months on different surfaces in some studies [29-32]. Trials in Tanzania with ICON 10 CS recorded 100% mortality of *An. gambiae *s.l. up to seven months on sprayed surfaces [33]. In Vietnam, persistence of effectiveness lasted up to four, five and three months, respectively, on wood, bamboo and brick walls in bioassays against *Anopheles dirus *[34]. In a WHOPES supervised trial with CS and WP formulations with 30 mg/m^2 ^in Benin, persistence of effectiveness was reported up to two months only, whereas the Indian trials reported persistence up to four-six months [34]. In India, IRS of ICON 10 CS formulation produced comparable or better efficacy than the WP formulation [29,34]. In the present study, ICON 10 CS formulation produced comparable or better performance than previously reported.

In comparison with the results presented by other authors, the current study revealed a clearly higher estimation of residual effect of deltamethrin WG 25%, bendiocarb WP 80% and lambda-cyhalothrin 10 CS on various surfaces against *An. gambiae *s.s. susceptible to all insecticides. Thus, deltamethrin WG and lambda-cyhalothrin CS may be used on the three types of walls, in areas where malaria vectors are still susceptible to pyrethroids, when applied in cycles every five-six months. Meanwhile bendiocard WP IRS, applied in cycles every trimester, would supplement long lasting insecticide treated nets in areas where vectors have developed resistance to pyrethroids.

## Conclusion

In view of the results, the evaluation of residual effects of bendiocarb WP 80%, lambda-cyhalothrin 10 CS and deltamethrin WG 25% on different indoor surfaces has established a baseline set of data that can be used for the re introduction of IRS in Cameroon. Any other candidate insecticide formulation to be used for IRS should be tested in real use conditions at community level so that the results would guide the decision makers on the spray cycles according to each type of indoor surface.

## Competing interests

The authors declare that they have no competing interests.

## Authors' contributions

JE designed the study, was responsible for the implementation and supervision of the study, analyzed and interpreted the data and drafted the manuscript. PN, JAM and MP participated in the implementation of the study, data collection and entry, as well as editing the manuscript. BM and DS assisted with the review of the study protocol, implementation of the study and editing the manuscript. PA assisted with the review of the study protocol, implementation of the study and edited the manuscript. All authors read and approved the final manuscript.
